# The Association of Polycystic Ovary Syndrome-Like Clinical Features and Socioeconomic Status on Health-Related Quality of Life

**DOI:** 10.1089/whr.2025.0008

**Published:** 2025-04-24

**Authors:** Stephanie Mohammed, Venkatesan Sundaram, Brian N. Cockburn, Shastri Motilal, Sasha Ottey, Ricardo Azziz

**Affiliations:** ^1^ Dept. of Preclinical Sciences, Faculty of Medical Sciences, The University of the West Indies at St Augustine, Republic of Trinidad and Tobago.; ^2^ Dept. of Physics, Faculty of Science and Technology, The University of the West Indies, St. Augustine, Republic of Trinidad and Tobago.; ^3^ Dept. of Basic Veterinary Sciences, School of Veterinary Medicine, Faculty of Medical Sciences, The University of the West Indies, St. Augustine, Republic of Trinidad and Tobago.; ^4^ Dept. of Life Sciences, Faculty of Science and Technology, The University of the West Indies, St. Augustine, Republic of Trinidad and Tobago.; ^5^ Dept. of Paraclinical Sciences, Faculty of Medical Sciences, The University of the West Indies, St. Augustine, Republic of Trinidad and Tobago.; ^6^ PCOS Challenge: The National Polycystic Ovary Syndrome, Atlanta, Georgia, USA.; ^7^ Dept. of Health Policy, Management, and Behavior, School of Public Health, University at Albany, SUNY, Rensselaer, New York, USA.; ^8^ Depts. of Obstetrics & Gynecology, Medicine, Heersink School of Medicine, University of Alabama at Birmingham, Birmingham, Alabama, USA.; ^9^ Dept. of Healthcare Organization & Policy, School of Public Health, University of Alabama at Birmingham, Birmingham, Alabama, USA.

**Keywords:** polycystic ovary syndrome, PCOS, socioeconomic status, SF-12, epworth sleepiness scale, Beck’s inventory, hirsutism, menstrual irregularities, quality of life

## Abstract

**Background:**

Polycystic Ovary Syndrome (PCOS) affects 8%–13% of reproductive-age women globally, with comorbidities including obesity, insulin resistance, type 2 diabetes, and psychological disorders. Socioeconomic status (SES) significantly impacts health outcomes.

**Methods:**

A community-based, pilot study was conducted in Trinidad among females aged 18–45 years, representing diverse ethnicities and SES. Participants underwent a standardized history and physical exam. Clinical hyperandrogenism (HIR) was assessed using the modified Ferriman–Gallwey scale (HIR ≥6), menstrual dysfunction (MD) as <9 cycles/year, depression *via* Beck’s Inventory, overall health using SF-12 v1, and daytime somnolence with the Epworth Sleepiness Scale. Data analysis included descriptive statistics, analysis of variance, and multinomial logistic regression adjusting for confounders.

**Results:**

Among 250 participants (mean age 31.6 ± 7.9 years), we classified 200 with clinical presentations, which included: no MD or HIR (56.7%), MD only (14.4%), HIR only (21.9%), and MD+HIR (7%). Age, income, and education were significantly correlated with clinical presentation. Older age reduced the risk of HIR (mean difference = 4.507, *p* = 0.004) and MD+HIR (mean difference = 9.063, *p* < 0.001). Income (OR = 0.37, 95% CI: 0.16–0.87, *p* = 0.022) reduced MD odds. Self-reported infertility was associated with MD (odds ratio [OR] = 0.27, 95% confidence interval [CI]: 0.11–0.65, *p* = 0.006). MD+HIR correlated with severe depression (OR = 5.96, 95% CI: 1.62–21.90, *p* = 0.007). Mental health scores (SF-12 MCS) were lower in women with MD+HIR (mean difference = −11.477, *p* = 0.005).

**Conclusion:**

Seven percent of women in this sample showed probable PCOS based on clinical manifestations, with SES impacting quality of life, mental health, and sleep. Higher income reduced MD and MD+HIR risk, while infertility increased MD risk and severe depression was linked to MD+HIR.

## Introduction

Polycystic ovary syndrome (PCOS) is a multifaced endocrine disorder with a spectrum of clinical features that can impact overall health. The World Health Organization estimates that PCOS affects 8%–13% of women of reproductive age, with approximately 70% of cases remaining underdiagnosed globally.^1^ The diagnosis and assessment of PCOS are guided by several established guidelines.^2–8^ Beyond its clinical manifestations, PCOS is associated with a range of comorbidities, including obesity, insulin resistance (IR), type 2 diabetes mellitus, cardiovascular disease (CVD) and psychological disorders such as depression and anxiety.^9–12^ Emerging evidence suggests that there is a strong association between PCOS and its components and the socioeconomic status (SES) of women.^13^

SES encompasses income, educational attainment, financial security and subjective perceptions of social status and social class. It affects overall human functioning, including our physical and mental health. Individuals with lower SES are at increased risk for engaging in adverse behaviors, including smoking, low physical activity, and poor nutritional routines.^14,15^ Sedentary lifestyle is linked to the increased prevalence of obesity, creating a strong association with SES.^16,17^

The link between SES and health outcomes is well-documented, yet its implications for women with PCOS remain underexplored. The interplay between SES and health-related quality of life (QoL), sleep patterns, and mental health is particularly significant given the multifaceted nature of PCOS and its associated comorbidities. Understanding how SES variables affect these dimensions can provide insight into the broader impacts of PCOS beyond the clinical manifestations and help identify targeted interventions. This approach aligns with recent calls to integrate socioeconomic factors into healthcare research to address disparities in care and outcomes.

The main objective of this study was to investigate the relationship between SES factors, QoL, sleep patterns, and mental health with PCOS-like clinical presentations, utilizing the SF-12, Epworth Scale, and Beck’s Scale. By focusing on these aspects, this study aims to shed light on how SES challenges intersect with the experiences of women with possible PCOS. Understanding these factors will not only assist in addressing disparities in healthcare access and treatment adherence but also contribute to developing comprehensive strategies for preventing, diagnosing, and managing PCOS. Integrating socioeconomic considerations into PCOS research is crucial for enhancing our understanding of the condition and ensuring equitable care for all affected individuals.

## Materials and Methods

This pilot study was a community-based, random sampling study conducted among females between the ages of 18 and 45 years throughout the eight major geographical locations in Trinidad from the period of January 2023 to August 2023. The areas within these locations were selected randomly (using a traditional method of placing all villages in the selected zone and then randomly selecting a village from that zone), and every 10th household was chosen for this study. Upon arrival, we requested if one female from the household between the ages of 18 and 45 years can speak with us. The participant was informed that this was a general reproductive assessment being conducted and if eligible the participant was selected for the interview to minimize bias. If the first individual did not meet the inclusion criteria, the next eligible female was selected. This process ensured that there was no preferential selection based on marital status, and the selection process was unbiased. In addition, we ensured that the households where both married and divorced women were selected were not influenced by any preconceived notions or social factors. Only one person was selected from each household. It is important to note that there may have been sampling bias because if some households were not available, the immediate household after was selected. Also, household with multiple women, may have over-represented who was available for the interview. The interview was conducted on the spot. Following this, participants were scheduled to meet at the Eric Williams Medical Sciences Complex, health centers close by and at pharmacies that provided space in the area to have their blood drawn and physical assessments. Ethical approval was obtained from the Campus Research Ethics Committee, The University of the West Indies, St. Augustine, Trinidad and Tobago (CREC-SA.1723/08/2022). The study is also listed under ClinicalTrials.gov with identification NCT05937360.

Inclusion criteria consisted of participants willingness to participate and consent to the study, women ages 18–45 years, and only one member of the household was selected randomly. Exclusion criteria were women less than 18 years or older than 45 years, family members within the same household, pregnant women and postmenopausal women, women who had undergone hysterectomy and/or bilateral oophorectomy, unwillingness to participate or difficulty understanding the consent process and anything that would subject the participant at increased risk or preclude the participants compliance with the study.

### Data collection

Participants were informed of the study and asked to provide both verbal and written consent. Data was then collected for each participant using a standard clinical report form (CRF) for uniform data collection. This information included socioeconomic data, Ferriman–Gallwey Score assessment,^18^ SF-12,^19,20^ Beck depression inventory,^21,22^ and Epworth Sleepiness Scale,^23^ assessments. Each CRF was assigned a serial identification number. Anthropometric measurements (weight, height, waist, and hip circumference) and a physical examination for hirsutism (HIR), acne, alopecia, acanthosis nigricans (AN) were conducted.

### Instruments and measures

Study subjects were divided into four subgroups according to their clinical presentation: menstrual dysfunction (MD) only, hirsutism (HIR) only, MD + HIR, and neither MD nor HIR (CONTROLS). We defined HIR using the modified Ferriman–Gallwey instrument with a cut of 6 or above.^18,24^ MD was defined as menstrual period lengths more than 35 days less than 21 days, or less than 9 cycles/year. Depression was categorized according to Beck’s inventory,^21,22^ as follows: No depression is awarded 0–4 points; minimal 5–7 points; moderate 8–15 points and severe depression ≥16. Dichotomous depression categories were created for no depression versus some level of depression and, severe depression versus non-severe depression.

The Epworth Sleepiness Scale,^23^ was used to score daytime somnolence with a positive score of >10. Obesity was classified based on body mass index (BMI) (kg/m^2^) with categories of underweight = <18.5, normal weight = 18.5–24.9, overweight = 25–29.9, and obesity >30. The SF-12 v1 questionnaire was used to assess QoL with scoring of the tool in keeping with developer’s recommendations (a validated survey developed by Ware et al.^25^ and licensed through Qualtrics,^26^ for the scoring algorithm.^20^ In the absence of Trinidad and Tobago norms, the SF-12 derived mental component score (MCS) and physical health outcome measures (PCS) were normalized to the U.S. population to a mean of 50 and a standard deviation (SD) of 10. Based on these normalized scores, a value greater than 50 indicated better QoL while values less than 50 indicated worse QoL.

The assessment of acne was made using a standard acne lesion assessment.^27^ Acne was scored according to whether it was grade 1 (mild), 2 (moderate), or 3 (severe). Mild acne was characterized by the presence of few to several papules and pustules, but no nodules. Moderate acne by the presence of several to many papules and pustules, along with a few to several nodules. Severe acne by the presence of numerous or extensive papules and pustules, as well as many nodules.

**FIG. 1.**
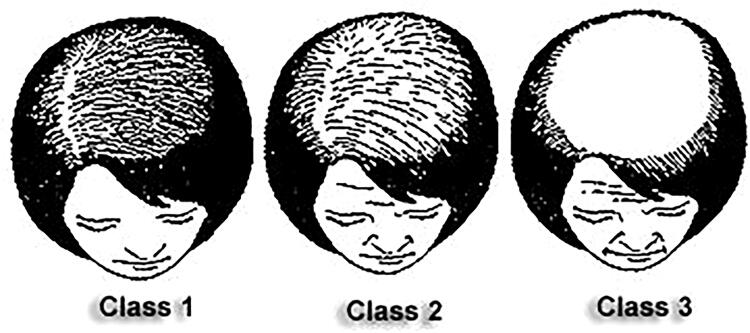
Ludwig scale for scoring androgenic alopecia in females. **Class 1**. In this stage, hair loss is considered mild. Such hair loss is noticeable when the hair is parted down the center of the scalp, as more and more scalp will become visible over time. **Class 2**. Type II hair loss is considered moderate and presented by thinning, shedding, a general decrease in volume, and a center part that continues to widen over time. **Class 3**. Type III is the final and most extreme classification of female hair loss.

The Ludwig Scale,^28,29^ (see Fig. 1) was used to diagnose the severity of female hair loss. From left to right in the image, these types include *Class I*, *II*, and *III*.

### Statistical analysis

Descriptive data were depicted using frequencies and proportions for categorical variables and means or medians for continuous data. Comparison of ordinal scores was done using analysis of variance with post hoc Bonferroni adjusted *p* values to reduce type-1 error inflation. Multinomial logistic regression was used to explore predictors of the three different clinical presentations (HIR only, MD only, and MD+HIR) with those without MD or HIR (*i.e.,* CONTROLS) as the referent outcome. Binary logistic regression was used to explore predictors of dichotomous variables. A *p* value of less than 0.05 was deemed statistically significant. Health outcomes from the SF-12 v1 were calculated using the scoring algorithm provided by Qualtrics.^25^ A *p* value of less than 0.05 was deemed statistically significant.

## Results

Of 400 participants approached, 250 agreed to participate in this study (response rate of 62.5%). As shown in the Table 1 the mean age of women was 31.6 years (SD 7.9) ranging from 18–45 years. The income categories in this study were assessed by monthly income (1 USD equates to approximately 7 [Trinidad and Tobago dollar [TTD]): “≤$5000 TTD” (53.7%), “$5001-$10,000 TTD” (28.9%), “$10,001–$15,000 TTD” (11.8%), “$15,001–$20,000 TTD” (3.3%), “$20,001–$25,000 TTD” (0.8%), and “≥$25,000 TTD” (1.6%). Marital status in this study was categorized as single, divorced, or separated (53.6%) and married or cohabiting (37.6%). The highest education level attained was primary/secondary (37.9%) and tertiary/vocational (62.1%). Ethnic distribution was East Indian (50.8%), African (14.5%), and Mixed/Other (34.7%). The mean BMI was 28.26 ± 9.13 kg/m^2^. The mean age of menarche was 12.19 ± 1.58 years. Among those with pregnancies, 57.1% had no pregnancies, 15.3% had one, 13.8% had two, and 13.8% had three or more. The mean age of first pregnancy was 24.12 ± 5.42 years. Regarding reproductive health, infertility was reported in 21.0% of respondents. Current contraceptive use was reported by 24.2% of participants. For clinical features, alopecia was absent in 72.4% of respondents, while 21.6% had mild alopecia and 6.0% had more severe presentations. Acne severity was classified as none (49.4%), mild (22.7%), moderate (15.0%), and severe (13.0%).

Of the 250 respondents, 49 (20%) participants declined to give information regarding menstrual cycle duration. These respondents were excluded from further analyses as they could not be classified as belonging to any of the four categories. Based on the National Institutes of Health (NIH) 1990 criteria, 56.7% of participants had neither HIR nor MD. HIR alone was present in 21.9% of cases, while 14.4% had MD only. A smaller proportion, 7.0%, exhibited both HIR and MD.

Data for Table 1, age, and income were the socio-demographic significantly associated with the PCOS clinical criteria. In additional analyses, higher age was associated with decreased odds of HIR only (OR = 0.923, 95% CI: 0.879–0.969, *p* = 0.001) and MD+HIR (OR = 0.831, 95% CI: 0.753–0.918, *p* < 0.001), but there was no significant association with the odds of MD only (OR 0.974, 95% CI: 0.922–1.028, *p* = 0.339). BMI, education, marital status, and ethnicity were not significantly related to clinical criteria. Those who earned more than $5,000 TTD per month were less likely to report MD (OR = 0.37, 95% CI: 0.16–0.87, *p* = 0.022) or MD+HIR. (OR = 0.19, 95% CI: 0.05–0.72, *p* = 0.015)

**Table 1.** Clinical Presentation and its Associations with Socio-demographic Characteristics and Reproductive Variables

Socio-demographics	CONTROLS	HIR only	MD only	MD+HIR	*p* value
Age, years (mean ± SD)	33.35 ± 7.20	28.84 ± 7.76	31.86 ± 7.87	24.29 ± 6.24	<0.001
Income $TTD, *n* (%)					
< $TTD5000	47 (41.2%)	19 (43.2%)	19 (65.5%)	11 (78.6%)	0.010
>$TTD5000	67 (58.8%)	25 (56.8%)	10 (34.5%)	3 (21.4%)	
Marital status, *n* (%)					
Single, divorced, or separated	61 (56.0%)	33 (76.7%)	14 (50.0%)	9 (64.3%)	0.070
Married or cohabiting	48 (44.0%)	10 (23.3%)	14 (50.0%)	5 (35.7%)	
Education, *n* (%)					
Primary/Secondary	32 (29.1%)	14 (32.6%)	15 (51.7%)	4 (30.8%)	0.148
Tertiary/ Vocational	78 (70.9%)	29 (67.4%)	14 (48.3%)	9 (69.2%)	
Ethnicity, *n* (%)					
East Indian	58 (50.9%)	21 (47.7%)	16 (55.2%)	8 (57.1%)	0.692
African	17 (14.9%)	10 (22.7%)	4 (13.8%)	0 (0.0%)	
Mixed/Other	39 (34.22%)	13 (29.5%)	9 (31.0%)	6 (42.9%)	
BMI, kg/m^2^ (mean ± SD)	27.69 ± 9.52	26.63 ± 6.94	30.83 ± 8.35	27.39 ± 11.86	0.322
Age of menarche, years (mean ± SD)	12.11 ± 1.51	12.27 ± 1.30	12.59 ± 1.86	13.21 ± 1.96	0.062
No. of pregnancies					
* *0	62 (54.4%)	31 (72.1%)	15 (51.7%)	11 (78.6%)	0.075
* *1 or more	52 (45.6%)	12 (27.9%)	14 (48.3%)	3 (21.4%)	
Age of first pregnancy, years (mean ± SD)	24.09 ± 5.09	27.17 ± 5.32	24.38 ± 4.70	19.67 ± 3.51	0.101
Infertility, *n* (%)					
* *Yes	17 (15.0%)	5 (11.6%)	11 (39.3%)	4 (28.6%)	0.011
* *No	96 (85.0%)	38 (88.4%)	17 (60.7%)	10 (71.4%)	
Contraceptive use, *n* (%) (current)					
* *Yes	22 (19.5%)	15 (34.1%)	9 (31.0%)	5 (35.7%)	0.154
* *No	91 (80.5%)	29 (65.9%)	20 (69.0%)	9 (64.3%)	
Alopecia score					
* *0	84 (73.7%)	29 (65.9%)	22 (75.9%)	10 (71.4%)	0.810
* *1	23 (20.2%)	12 (27.3%)	7 (24.1%)	3 (21.4%)	
* *2	7 (6.1%)	3 (6.8%)	0 (0%)	1 (7.1%)	
Acne score					
None (0)	67 (58.8%)	15 (34.1%)	10 (34.5%)	3 (21.4%)	0.001
Mild (1)	23 (20.2%)	9 (20.5%)	11 (37.9%)	2 (14.3%)	
Moderate (2)	14 (12.3%)	9 (20.5%)	6 (20.7%)	3 (21.4%)	
Severe (3)	10 (8.8%)	11 (25.0%)	2 (6.9%)	6 (42.9%)	

HIR, hirsutism; MD, menstrual dysfunction; TTD, Trinidad and Tobago dollar; BMI, body mass index.

**FIG. 2.**
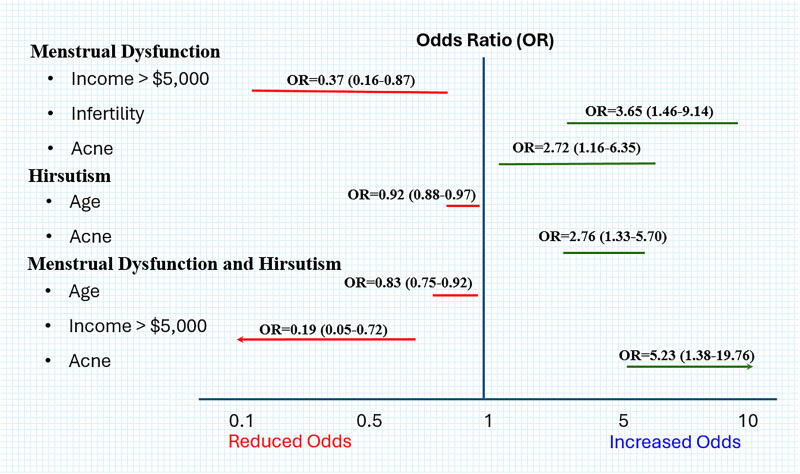
Significant socio-demographic predictors of clinical presentation, including menstrual dysfunction and/or hirsutism.

With regards to reproductive variables only self-reported infertility and acne were significantly associated with clinical presentation. While there were no significant differences between the rates of infertility for women with HIR only (*p* = 0.585) or those with MD+HIR (*p* = 0.208), women with MD only had significantly increased odds of infertility (OR = 3.65, 95% CI: 1.46–9.14, *p* = 0.006) when compared to CONTROLS. With respect to acne, the highest prevalence of severe acne was seen in the MD+HIR group at 43%. Compared to the controls, those with MD+HIR had significantly increased odds of reporting severe acne compared to those without acne (OR = 13.4, 95% CI: 2.88–62.32, *p* = 0.001). Neither age of menarche, age of first pregnancy, contraceptive use nor alopecia were associated with either clinical presentation. Figure 2 depicts the significant associations between the above socio-demographic and clinical variables that were significantly associated with the three main clinical presentations when compared to those with neither.

### Clinical presentation as a predictor of daytime sleepiness and depression

In this study, Beck’s depression categories were no depression (27.5%); minimal depression (15.7%); moderate depression (31.4%); and severe depression (25.4%). With regards to the Epworth sleepiness scale, 22.4% of participants had daytime somnolence. From the SF-12 tool, the mean PCS score was 41.50 (SD = 8.49) and the MCS score was 45.16 (SD = 10.80). Of the sample, 86.5% had a PCS score of less than 50 while 64.4% had an MCS score of less than 50.

Table 2 depicts the association of clinical presentation with depression and daytime sleepiness. MD+HIR was significantly associated with the severe depression category compared to non-severe depression after adjustment for demographic confounders. There was no such association when the other combined levels of depression (minimal, moderate, and severe) were compared to those with no depression.

**Table 2.** Association of Clinical Presentation with Daytime Somnolence and Depression Levels

Clinical presentation	HIR only aOR (95% CI)^a,b^	*p* value	MD only aOR (95% CI)^a,b^	*p* value	MD+HIR aOR (95% CI)^a,b^	*p* value
Daytime sleepiness	1.21 (0.51–2.88)	0.664	2.22 (0.87–5.66)	0.095	1.42 (0.37–5.49)	0.608
Severe depression	1.29 (0.52–3.16)	0.584	2.32 (0.90–6.00)	0.081	5.96 (1.62–21.90)	0.007
Any depressive category	2.14 (0.87–5.27)	0.097	2.30 (0.78–6.76)	0.131	1.56 (0.37–6.55)	0.540

^a^ Adjusted for age, income, and education level.

^b^ Referent criteria have neither HIR or MD.

HIR, hirsutism; MD, menstrual dysfunction; aOR, adjusted odds ratio; CI, confidence index.

### Clinical presentation as a predictor of global mental and physical health outcomes

The mean SF-12 derived (SD) PCS, and MCS were 41.5 (8.5) and 45.2 (10.8), respectively. Table 3 depicts the association between the clinical presentation of PCS and MCS health scores. There were no associations between clinical presentation and worse health outcome states (scores less than 50) for both PCS and MCS. Zero frequencies in the composite category precluded odds ratio calculation. In additional linear models, there were no associations between the four clinical presentations and PCS score (mean square = 41.372, df = 3, F = 0.605, *p* = 0.613) after adjustment for age, income, and education level. There was, however, a significant association between the clinical presentation and the MCS score (mean square of 574.178 [df = 3], F = 5.525, *p* = 0.001). The estimated marginal means for MCS score, adjusted for age at 31.36, were 46.702 for CONTROLS (95% CI: [44.412, 48.993]), 45.678 for HIR only (95% CI: [42.353, 49.002]), 40.303 for MD only (95% CI: [36.175, 44.431]), and 35.226 for MD+HIR (95% CI: [28.895, 41.557]).

**Table 3.** Association of Clinical Presentation with Mental and Physical Health Scores

Clinical presentation	Worse physical health (lower PCS) aOR (95% CI)^a^	*p* value	Worse mental health (lower MCS) aOR (95% CI)^a^	*p* value
CONTROLS^b^	1		1	
HIR only	1.07 (0.38–3.044)	0.888	0.98 (0.44–2.17)	0.951
MD only	2.24 (0.46–10.95)	0.317	2.34 (0.79–6.96)	0.124
MD+HIR	^c^	0.999	^c^	0.999

^a^ Adjusted for age, income, and education level.

^b^ Referent criteria.

^c^ Odds ratio incalculable due to 0 frequencies.

HIR, hirsutism; MD, menstrual dysfunction; aOR, adjusted odds ratio; CI, confidence index; PCS, physical component score.

Post-hoc contrasts confirmed statistically significant differences. The MD only subgroup scored significantly lower than CONTROLS (mean difference = −6.400, *p* = 0.047). Similarly, the MD+HIR group scored significantly lower than either CONTROLS or the HIR only subgroup (mean difference = −11.477, *p* = 0.005; mean difference = −10.452, *p* = 0.022, respectively).

## Discussion

In this pilot study, 7% of participants have MD and HIR (probable PCOS). This mirrors the prevalence found in other settings where 6.5% of participants were diagnosed with PCOS.^30^

This study provides valuable insights into the interactions between PCOS-like clinical features, SES, and health status among women in Trinidad. Our findings underscore the significant impact of SES on the prevalence and severity of PCOS-related symptoms and their subsequent influence on mental and physical health outcomes.

This study reveals a clear association between socio-demographic factors, particularly age and income, and the clinical presentation of PCOS-like symptoms. Younger age was significantly associated with higher odds of HIR and the combined presence of MD and HIR, which aligns with the known pathophysiology of PCOS that often manifests during adolescence and early adulthood. In young women with PCOS, hyperandrogenism, menses irregularities, and IR may occur together, emphasizing the pathophysiological role of excess androgen and insulin on PCOS.^31^ Hyperandrogenism and infertility represent major complaints of PCOS in adult fertile age. Later in life, it becomes clear that the association of obesity (particularly the abdominal phenotype) and PCOS renders affected women susceptible to developing T2D, with some differences in prevalence rate among countries.^31^

The finding that higher income levels are associated with lower odds of experiencing MD and MD+HIR highlights the role of SES in modulating health outcomes. Women with higher education levels and more income may have access to oral contraceptives (OCPs) to regulate their menstrual cycles.^13^ This could reflect better access to healthcare, healthier lifestyle choices, and increased awareness of health issues among women in higher income brackets. These findings are consistent with global patterns, where socioeconomic disparities contribute to variations in the prevalence and management of PCOS and other health conditions.

This study also highlights the profound impact of PCOS-like symptoms on mental health, particularly in relation to severe depression. Women with both MD and HIR had significantly higher odds of severe depression compared to those without these symptoms, independent of SES. This suggests that the combination of these two clinical features may exacerbate the psychological burden associated with PCOS. PCOS can affect mental health through various physical manifestations of the disorder, such as obesity, infertility, stress, HIR, acne, and socio-demographic factors, all of which can lead to dissatisfaction with self-image in women.^32–40^ Patients with PCOS also experience anovulation and menstrual irregularities, which can lead to fertility difficulties and associated real or perceived social pressure, potentially resulting in depression and anxiety.^41^ Research has indicated an association between PCOS and both depression (27.5%) and anxiety (13.3%) among adults,^42^ and adolescents with PCOS have also been found to be at risk of depression.^43^ MD and HIR are strongly correlated with androgen excess and hyperinsulinism as well.^18,39,44^ The interplay between these physical manifestations and mental health outcomes underscores the necessity for a comprehensive care approach that addresses both the physical and psychological aspects of PCOS.

In addition, there is a social gradient in mental health, with higher levels of income inequality being linked to a higher prevalence of mental illness.^45^ Women with lower education have a higher risk of developing PCOS compared to their more educated counterparts.^46^ Those in insecure, low-status jobs are more susceptible to chronic stress, and low employment rank is a strong predictor of depression. Education level has been associated with increased risks of hypertension, CVD, and other comorbidities.^47–49^ Women’s perception of their income level and employment status were found to be statistically significant factors for depression symptoms (*p* = 0.033 and *p* = 0.003, respectively) with lower depression symptoms observed in women with PCOS who worked and perceived their income as sufficient.^50^ Moreover, there may be a link between PCOS-like symptoms and pro-inflammatory markers, contributing to depression.^51^ This dissatisfaction can further exacerbate the biochemical effects of androgen excess and abdominal obesity resulting in dyslipidemia and metabolic syndrome.^33^

Interestingly, while the study found significant associations between PCOS-like symptoms and mental health, the impact on physical health, as measured by the SF-12 PCS, was less pronounced in this study. This may suggest that the mental health effects of PCOS are more immediate or severe compared to its impact on physical health, or it could reflect limitations in the study’s ability to capture the full spectrum of physical health impacts. Nonetheless, the significant decrease in the MCS among women with MD and MD+HIR compared to controls highlights the importance of mental health as a critical aspect of overall well-being in women with PCOS-like symptoms. While the association between PCOS and mental health, has been well established, our study adds a layer of complexity by considering how socioeconomic factors influence these outcomes in women with probable PCOS-like symptoms. Given the cross-sectional nature of this study, we cannot infer causality between PCOS and mental health issues. However, the significant correlation between the presence of menstrual dysregulation and HIR (PCOS-like features) with severe depression in our cohort underscores the importance of mental health screening and support for women with these symptoms. These findings further highlight the need for comprehensive care that addresses both the physical and psychological impacts of PCOS-like symptoms, regardless of formal diagnosis.

The association between self-reported infertility and the presence of MD in our study is another critical finding. Women with MD had significantly higher odds of reporting infertility, which is a well-documented complication of PCOS.^52,53^ The burden of infertility attributable to PCOS increased from 6 million prevalent cases in 1990 to 12.13 million in 2019 globally and increased sharply in most regions and nations.^54^ This finding not only reinforces the reproductive challenges faced by women with PCOS but also underscores the importance of early diagnosis and intervention to manage infertility risks in this population.

The results of this study have important implications for both clinical practice and public health strategies.

The association between PCOS-like symptoms and health status seen in this study was independent of SES. This highlights the need for ongoing research on PCOS across all age and income groups. Tailored interventions that consider the socioeconomic context of patients may be more effective in improving both mental and physical health outcomes in women with PCOS. In addition, the high prevalence of severe depression among women with combined MD and HIR highlights the need for mental health screening and support as a routine part of PCOS care.

While this study provides significant insights, it is not without limitations. One key limitation of this study is the lack of formal verification of PCOS as we relied on clinical features such as MD and HIR to identify women with probable PCOS. Without a confirmed diagnosis, it is difficult to conclusively associate these features with PCOS. The use of the NIH criteria for identifying probable PCOS-like clinical features was a necessary step. Blood samples were collected this data is expected to change when we analyze them. In addition, the low sample size, though sufficient for exploratory analysis, should be interpreted with caution and requires a larger and more diverse sample size. The cross-sectional design precludes the ability to draw causal inferences, and the reliance on self-reported data for certain variables may introduce bias. In addition, the generalizability of the findings may be limited to similar populations, and further research is needed to explore these associations in diverse settings. However, it is important to note that participants were informed of this being a reproductive assessment and not a PCOS probable assessment which can control some of the bias experienced from over or under-reporting symptoms specifically related to PCOS. We also included persons on OCPs to provide a comprehensive view of the health status of women from our preliminary data. The association between income and PCOS-like symptoms warrants further clarification also as higher income and education levels are often linked to better access to healthcare, including combined hormonal contraceptives, which can potentially regulate the menstrual cycle. This might confound certain aspects of reproductive health assessment, particularly regarding natural cycle irregularities that are common in PCOS. Despite this, we utilized the NIH PCOS diagnostic criteria which does not exclude women on hormonal contraceptives. Future studies could benefit from longitudinal designs that track the progression of PCOS symptoms and related health outcomes over time, as well as interventions aimed at mitigating the impact of SES on PCOS.

## Conclusion

In conclusion, this pilot study contributes to the growing body of literature on the role of socioeconomic factors in the health outcomes of women with probable PCOS. By highlighting the interactions between SES and PCOS-like symptoms, our findings emphasize the need for holistic, socially informed approaches to the management and treatment of PCOS. These insights can help guide more equitable health care practices and improve the QoL for women affected by this condition.

Seven percent of our medically unbiased population in Trinidad demonstrated clinically evident probable PCOS (MD+HIR) fits with international rates using the NIH criteria. PCOS was associated with severe depression and impaired mental QoL even after adjustments for socioeconomic factors. This lends justification for further research and intervention across all age and economic groups. Integrating SES considerations into PCOS research and clinical practice is crucial for equitable healthcare.

## Data Availability

The data that support the findings of this study are available from the corresponding author (S.M.), upon reasonable request.

## Abbreviations Used

CRF: Clinical report form
CVD: Cardiovascular disease
HIR: Hirsutism
IR =: Insulin resistance
MD: Menstrual dysfunction
OCPs: Oral contraceptives
OR: Odds ratio
PCOS: Polycystic ovary syndrome
SD: Standard deviation
SES: Socioeconomic status
T2DM: Type 2 diabetes mellitus
