# Supplementary material for: The Association of Polycystic Ovary Syndrome-Like Clinical Features and Socioeconomic Status on Health-Related Quality of Life
Source: Womens Health Rep (New Rochelle). 2025 Apr 24;6(1):493–503. doi: 10.1089/whr.2025.0008 (PMC12177319; doi:10.1089/whr.2025.0008)
Supplement: Supplementary Table S1 [file whr.2025.0008_supplementary_table_s1.docx]

Supplementary

| **Variable** | **(n(%)** |
| --- | --- |
| **Age, years (mean ± SD), n=241** | 31.56 ± 7.91 |
| **Income (TT$), n=246** |  |
| < 5,000 | 132 (53.7%) |
| 5,001-10,000 | 71 (28.9%) |
| 10,001-15,000 | 29 (11.8%) |
| 15,001-20,000 | 8 (3.3%) |
| 20,001-25,000 | 2 (0.8%) |
| >25,000 | 4 (1.6%) |
| **Education, n=232** |  |
| Primary/Secondary | 88 (37.92%) |
| Tertiary/Vocational | 144 (62.1%) |
| **Ethnicity, n=248** |  |
| East Indian | 126 (50.8%) |
| African | 36 (14.5%) |
| Mixed/Other | 86 (34.7%) |
| **Marital Status, n=228** |  |
| Single/Divorced/Separate | 134 (53.6%) |
| Married/Cohabiting | 94 (37.6%) |
| **BMI, kg/m² (mean ± SD), n=235** | 28.26 ± 9.13 |
| **Age of Menarche, years (mean ± SD), n=245** | 12.19 ± 1.58 |
| **Number of Pregnancies, n=240** |  |
| 0 | 137 (57.1%) |
| 1 | 37 (15.3%) |
| 2 | 33 (13.8%) |
| 3 or more | 33 (13.8%) |
| **Age of First Pregnancy, years (mean ± SD), n=95** | 24.12 ± 5.42 |
| **Infertility, n=238** |  |
| Yes | 50 (21.0%) |
| No | 188 (79.0%) |
| **Contraceptive Use, n=240** |  |
| No | 182 (75.8%) |
| Yes | 58 (24.2%) |
| **Alopecia Score, n=250** |  |
| 0 | 181 (72.4%) |
| 1 | 54 (21.6%) |
| 2 | 15 (6.0%) |
| **Acne Score, n=247** |  |
| None | 122 (49.4%) |
| Mild | 56 (22.7%) |
| Moderate | 37 (15.0%) |
| Severe | 32 (13.0%) |
| **Age, years (mean ± SD), n=241** | 31.56 ± 7.91 |

*Table X – Socio-demographics and clinical variables of respondents*
